# Nutraceutical Interventions for Post-Traumatic Stress Disorder in Animal Models: A Focus on the Hypothalamic–Pituitary–Adrenal Axis

**DOI:** 10.3390/ph15070898

**Published:** 2022-07-20

**Authors:** Mudan Cai, Hee Ra Park, Eun Jin Yang

**Affiliations:** Department of KM Science Research Division, Korea Institute of Oriental Medicine (KIOM), 1672 Yuseong-daero, Yuseong-gu, Daejeon 34054, Korea; mudan126@kiom.re.kr (M.C.); hrpark0109@kiom.re.kr (H.R.P.)

**Keywords:** post-traumatic stress disorder, hypothalamic–pituitary–adrenal axis, herbal medicine, glucocorticoid, neurotransmitter

## Abstract

Post-traumatic stress disorder (PTSD) occurs after exposure to traumatic events and is characterized by overwhelming fear and anxiety. Disturbances in the hypothalamic–pituitary–adrenal (HPA) axis are involved in the pathogenesis of mood disorders, including anxiety, PTSD, and major depressive disorders. Studies have demonstrated the relationship between the HPA axis response and stress vulnerability, indicating that the HPA axis regulates the immune system, fear memory, and neurotransmission. The selective serotonin reuptake inhibitors (SSRIs), sertraline and paroxetine, are the only drugs that have been approved by the United States Food and Drug Administration for the treatment of PTSD. However, SSRIs require long treatment times and are associated with lower response and remission rates; therefore, additional pharmacological interventions are required. Complementary and alternative medicine therapies ameliorate HPA axis disturbances through regulation of gut dysbiosis, insomnia, chronic stress, and depression. We have described the cellular and molecular mechanisms through which the HPA axis is involved in PTSD pathogenesis and have evaluated the potential of herbal medicines for PTSD treatment. Herbal medicines could comprise a good therapeutic strategy for HPA axis regulation and can simultaneously improve PTSD-related symptoms. Finally, herbal medicines may lead to novel biologically driven approaches for the treatment and prevention of PTSD.

## 1. Introduction

Post-traumatic stress disorder (PTSD) is a mental disorder that occurs after exposure to traumatic events, such as natural disasters, warfare, traffic collisions, child abuse, rape, and witnessing injuries or death. PTSD is accompanied by several symptoms, including negative thoughts, avoidance or numbing, re-experiencing, and hyperarousal; these last for months or years [1]. In the United States, approximately 3.5% of adults are diagnosed with PTSD every year, and the prevalence of PTSD is twice as high in women than in men [1]. The pre-existing risk factors for the development of PTSD are both environmental and genetic in nature. Due to population heterogeneity and individual differences (both genetic and environmental), many studies have identified valid markers through gene × environment (G × E) studies. The genetic risk factors for PTSD include several genes, such as the solute carrier family 6 member 4 gene (*SLC64A*), FK506 binding protein 5 gene (*FKBP5*), gamma-aminobutyric acid (GABA) A receptor alpha 2 gene (*GABRA2*), a regulator of G-protein signaling 2 gene (*RGS2*), and Cordon-bleu WH2 repeat protein gene (*COBL*) [2]. FKBP5 is an important co-chaperone that regulates glucocorticoid receptor (GR) sensitivity; it has been shown to disrupt the binding between GRs and cortisol [3]. Furthermore, the physiological mechanism of PTSD involves disruption of the hypothalamic–pituitary–adrenal (HPA) axis and alteration of inflammation, neurotransmission, and neurotropic function [4]. These chronic changes result in many psychiatric phenotypes. Moreover, PTSD is closely associated with depression, anxiety, alcohol addiction, insomnia, and body pain. Therefore, preclinical studies have evaluated the efficacy of drugs against PTSD using behavioral tests designed for the assessment of memory, anxiety, and depression.

Currently, the major treatments for PTSD comprise anxiolytics and antidepressants. Furthermore, the only United States Food and Drug Administration-approved drugs for the treatment of PTSD are the following selective serotonin reuptake inhibitors (SSRIs): sertraline and paroxetine. SSRIs comprise the first-line treatment for psychiatric disorders, including depression and anxiety. However, there are some limitations to SSRI-based treatments, such as a long treatment time, and lower response and remission rates. Cognitive-processing therapy (CBT), a form of evidence-based psychotherapy, has greatly affected the treatment of PTSD; however, the non-response rates for CBT are as high as 50% in patients with PTSD [5]. Therefore, effective treatments for patients are limited, and more pharmacological interventions are needed [6,7].

Complementary and alternative medicine (CAM) has been used worldwide for the treatment of mental disorders; CAM includes herbal medicines, acupuncture, massage, yoga, and relaxation therapy. Among these, herbal medicines contain many active compounds that can target several mechanisms of disease pathology. Therefore, traditional medicines and their active compounds may be potential therapeutic candidates for mental disorders. Numata et al. demonstrated that 2 weeks of treatment with saikokeishikankyoto (Chaihu-Guizhi-Ganjiang-Tang in Chinese) significantly improved the “Impact of Event Scale–Revised” scores of patients with PTSD following the Great East Japan earthquake and tsunami [8]. After the coronavirus disease 2019 (COVID-19) pandemic, the Lily Bulb, Rehmannia Decoction, and Guilu Erxian Decoction were effectively used to treat COVID-19-induced PTSD [9]. Furthermore, many studies have demonstrated that herbal medicines regulate gut dysbiosis [10], insomnia [11], chronic stress [12], and depression [13] via HPA axis regulation.

Therefore, in this review, we briefly describe how PTSD disturbs the HPA axis through molecular pathological changes in PTSD-related brain regions. We also summarize the possibility of treating PTSD with herbal medicines and the cellular and molecular mechanisms underlying the same in animal PTSD models.

## 2. Methods

We screened 20 English electronic databases for articles on the efficacy of herbal medicines for PTSD. Among these databases, the PubMed database was screened for relevant articles published until May 2022 using the following MeSH terms: “Stress Disorders, Post-Traumatic” [MeSH terms]) AND herbal medicine (“herbal medicine” [MeSH Terms]) AND animal study (“animals” [MeSH terms]). Articles on animal models with footshock- and single prolonged stress (SPS)-induced PTSD were included.

## 3. Role of the HPA Axis in Stress Response

Many studies have demonstrated a relationship between the HPA response and stress vulnerability. Once the axis is activated, the corticotropin-releasing hormone (CRH) is released from the paraventricular nucleus (PVN) of the hypothalamus. CRH further activates the release of the adrenocorticotropic hormone (ACTH) from the pituitary gland. ACTH in turn promotes the release of glucocorticoids (cortisol in humans and corticosterone (CORT) in rodents) from the adrenal cortex. The released glucocorticoids rapidly bind to the GR and the mineralocorticoid receptor (MR), which translocate to the nucleus to regulate gene transcription. The activities of the MR and GR differ in specific regions; accordingly, the released glucocorticoids exert different physiological effects [14]. The GR is ubiquitously expressed in the body and is related to energy distribution (glycogenesis) and immune function; moreover, it is highly expressed in the hippocampus, amygdala, prefrontal cortex, and PVN. Conversely, the MR is mainly expressed in the limbic areas, especially the hippocampus.

Glucocorticoids mediate negative feedback and regulate the HPA axis to inhibit the stress response; this involves inhibition of ACTH release from the pituitary gland and CRH release from the hypothalamus and hippocampus. CRH overexpression results in impaired negative feedback (pexacerfont, a CRH-1 receptor antagonist, was developed for the treatment of anxiety and stress-related disorders [15,16]). High levels of CRH have been found in the cerebrospinal fluid of victims of suicide [17]. Furthermore, increased CRH levels have been observed to be associated with Alzheimer’s disease and major depression [18]. Therefore, an abnormally enhanced activity of the HPA axis fails to terminate the stress response, leading to psychiatric disorders (such as PTSD, schizophrenia, depression, and anxiety disorders).

## 4. Role of the HPA Axis in PTSD

Previous studies have demonstrated that the heterogeneity of PTSD leads to diverse symptoms (such as fear memory, anxiety, depression, and insomnia) through several neurobiological mechanisms, including HPA axis activation, immune system activation, alteration of the neurotransmitter system, and abnormal neural circuits [19]. For two decades, disturbances in the HPA axis have been considered the main mechanism for psychiatric disorders, especially the stress response. D’Elia et al. found that the plasma ACTH and salivary cortisol levels were higher in patients with PSTD developed after sexual assault compared to those controls with no history of trauma [20].

SPS and inescapable shock-induced models are well-known animal models for PTSD [21,22,23]. In SPS-induced PTSD animal models, increased plasma levels of CORT, CRH, and ACTH are associated with memory impairment due to dysregulation of the HPA axis [24,25]. Furthermore, animal studies have strongly implicated the role of CRH in fear-related behaviors; CRH regulates the activation of the noradrenergic and dopaminergic systems [25,26]. Moreover, the 5-HT concentration is reduced and the monoamine oxidase A (MAO-A) activity and the 5-hydroxyindoleacetic acid (5-HIAA)/5-HT ratio increased in the hippocampi of SPS-induced PTSD animal models [27]. Under stress conditions, glucocorticoids increase MAO-A in the brain through the stimulation of the Kruppel-like factor-11 (KLF11) and the cell-division cycle-associated 7-like protein pathways [28]. Homberg suggested that there is a dose-dependent U-shaped relationship between 5-HT levels and conditioned fear [29]. Therefore, regulating the 5-HT levels through SSRIs and MAO-A inhibitors may help treat PTSD. Furthermore, blocked reconsolidation of fear memory and facilitation of fear-memory extinction are key to the development of therapies for PTSD [30]. Drugs may interfere with reconsolidation and memory extinction. Quervain et al. suggested that glucocorticoids reduce fear-memory retrieval or enforce fear-memory extinction. Therefore, glucocorticoid-based interventions might be useful strategies for fear-related disorders [31]. Moreover, glucocorticoids are also related to the initial consolidation and reconsolidation of fear memory in a dose-dependent, inverted U-shaped manner [32].

### 4.1. Changes in the HPA Axis in PTSD-Related Brain Regions

Using magnetic resonance imaging and positron emission tomography, numerous studies have indicated decreases in the volumes and functions of the hippocampus, amygdala, and medial prefrontal cortex (mPFC) in patients with PTSD [33,34,35,36,37]. Therefore, alterations in the neurocircuits of the hippocampus, amygdala, and mPFC on brain imaging are closely associated with PTSD pathophysiology. Moreover, neuroanatomical changes regulate the activation of the HPA axis in response to stress in the brain [38].

The hippocampus is the main region of the brain associated with learning and memory. Subregions of the hippocampus (Cornu Ammonis (CA)1, CA3, and the dentate gyrus) play distinct roles in the encoding and processing of memories, which are disrupted in PTSD. It has been reported that the subregions of the hippocampus are smaller in patients with PTSD [33,39,40]. Chronic glucocorticoid exposure induces hippocampal atrophy, possibly through losses in the dendrites and synapses [41]. A prolonged glucocorticoid-induced reduction in the hippocampal volume may cause memory deficits (encoding, consolidation, retrieval, extinction, and reconsolidation); in particular, hippocampal CA3 lesions induce memory retrieval deficits that are blocked by adrenocortical suppression [42]. This means that glucocorticoids serve as sensors of stress responses in the hippocampal region. The binding of the hippocampal MR and GR regulates elevated levels of glucocorticoids through an indirect HPA axis activation [43,44]. Chronic glucocorticoid exposure reportedly changes the length of the neuronal dendrites and the number of the dendritic branch points; furthermore, it also decreases the synapse number and glial volume in the hippocampus [41]. In addition, glucocorticoids that are directly injected into the hippocampus induce structural changes, such as cell-layer irregularities, dendritic atrophy, soma shrinkage and condensation, and nuclear pyknosis [45]. Furthermore, PTSD is also associated with synaptic impairment, which enhances neuronal apoptosis through caspase activation in the hippocampus [46]. The brain-derived neurotrophic factor (BDNF) is essential in fear conditioning and fear extinction in the brain. Treatment with dexamethasone, a GR agonist, reportedly decreased BDNF mRNA expression in mice hippocampal BZ cells [47]. Interestingly, Cheng et al. found that the GR expression increased in the hippocampal dentate gyrus and decreased in the basolateral amygdala in an SPS-induced PTSD animal model. Furthermore, the neuroligin (NLG)-1/NLG-2 ratio, which is a measure of the postsynaptic activity, is increased in the hippocampus but decreased in the amygdala [48]. Wang et al. found that WAY-100635, a 5-HT1A receptor inhibitor, partially inhibited an SPS-induced increase in the expression of the GR (in the hippocampus) and CRH (in the hypothalamus) [49]. Thus, targeting the HPA axis and the serotonin system in the hippocampus is associated with modulating the HPA axis response in PTSD.

The emotions of fear, threat, and aggression are directly related to the amygdala region of the brain and anatomically connected to the hippocampus. An increasing number of studies on PTSD have focused on the basolateral complex of the amygdala (BLA; this includes the basal, lateral, and accessory basal nuclei) and the central nucleus (Ce). An overactive BLA (facilitating fear memory) or a dysfunctional Ce (failing to remove a fear memory) are related to the symptoms of PTSD [50]. Roozendaal et al. found that the growth of the dendritic spines affects abnormalities in the amygdala structure and function in severe stress-induced chronic anxiety [51]. Moreover, Neves et al. demonstrated a footshock-induced increase in the proximal dendritic spine density of BLA neurons in a rat PTSD model [52]. Furthermore, animal studies also demonstrated that delayed spinogenesis and synaptic transmission in the BLA increased anxiety-related behavior after PTSD [53]. Moreover, the effect of glucocorticoids on memory consolidation is critically dependent on BLA activation [54,55]. Using an SPS-induced PTSD animal model, Han et al. demonstrated that the levels of the MR and GR first decreased in the amygdala after 1 day of SPS and then increased gradually 14 days later [56]. Furthermore, the elevated plus maze (EPM) test has previously revealed that GR and MR antagonists increase the anxiety response [57]. Kim et al. demonstrated that electrolytic lesions or inactivation in the amygdala prevent the stress-induced impairment of hippocampal long-term potentiation and spatial memory [58,59]. These results suggest that amygdalar neuronal activity is necessary for stress-induced decreases in synaptic strength and memory. Furthermore, the BLA neurons project directly to the nucleus accumbens (NAc) to modulate memory consolidation; the NAc also receives direct projections from the hippocampus. Roozendaal et al. demonstrated that the BLA-NAc circuit plays an important role in mediating the effects of glucocorticoids on memory consolidation [60]. Inhibition of BDNF signaling in the amygdala impairs both the acquisition and consolidation of fear conditioning and the consolidation of fear memory extinction [61].

The PFC also plays an important role in working memory and memory formation and retrieval, particularly in terms of fear memory. Neuroanatomically, the mPFC–amygdala circuit is relevant to PTSD development. PFC destruction results in an increase in amygdala-related behaviors, such as fear conditioning and emotional learning [62,63]. This indicates that the BLA and PFC are partners in fear learning and extinction. Generally, fear responses regulate the acquisition and consolidation of fear memory through the mPFC–BLA–Ce–brainstem/hypothalamic circuit [64]. Studies have found that the mPFC inhibits impulse transmission from the BLA to the Ce through GABAergic intercalated cells [65,66]. In addition, chronic stress-induced increases in glucocorticoids significantly decrease the apical spine density and length in the mPFC in rats. This indicates that the loss of dendrites and spines affects cellular functions and contributes to PFC dysfunction in PTSD. Licznerski found that the serum/glucocorticoid regulated kinase 1 (SGK1) expression was significantly reduced in the mPFC of patients with PTSD. In addition, SGK1 inhibition in rat mPFC resulted in helplessness-like behaviors in rodent models through dendritic spine loss and synaptic activity dysfunction [67]. In addition, Diorio et al. suggested that the mPFC is a target site for terminating the negative feedback effects of glucocorticoids on the HPA axis-related responses to stress [68]. Wen et al. demonstrated that the expression of GR, calreticulin (CRT), and protein kinase C (PKC) increased in the mPFC of SPS groups. The results suggest that altered CRT and GR/PKC-dependent pathways are involved in the mechanism of a dysfunctional mPFC region in PTSD [69].

These findings suggest that three regions of the brain, i.e., the hippocampus, amygdala, and mPFC are targets for glucocorticoid-mediated negative feedback effects on stress-induced HPA activity disturbance and that these effects are dependent on the vulnerability and severity of stress.

### 4.2. Involvement of the HPA Axis in Inflammation in PTSD

The immune response is related to the activation of the HPA axis; increased CRH and glucocorticoid levels induce the production of pro-inflammatory cytokines (IL-1 and TNF-α) and anti-inflammatory cytokines (IL-4 and IL-10) from monocytes and mast cells to maintain the body balance [70,71,72,73]. Furthermore, chronic high levels of glucocorticoids continuously activate the immune cells, resulting in glucocorticoid resistance in the adrenal glands under chronic stress conditions. Glucocorticoid resistance induces adrenal fatigue leading to immune dysfunction [74]. Additionally, the PFC is a brain region that is sensitive to inflammatory injury that can worsen on glucocorticoid usage [75]. Compared to controls, police officers with PTSD showed increased levels of the C-reactive protein (CRP) and IL-6 (which are inflammatory markers) [76]. The levels of IL-6, IL-1β, tissue necrosis factor α (TNF-α), and CRP have been reported to be elevated by maltreatment and parental separation in childhood [77]. In addition, the Il-1β and TNF-α levels are significantly higher in animal models and patients with PTSD [78]. Moreover, SPS activates TLR4/MyD88/NF-κB pathway-mediated inflammation in the heart, contributing to the pathogenesis of cardiac dysfunction in PTSD [79]. Therefore, inflammatory cytokines are important factors in PTSD development.

Astrocytes play an important role in the regulation of the immune response in the brain, which maintains brain homeostasis and neuronal metabolism. Recent studies have shown that the astrocyte density decreases, and the astrocyte morphology changes in the hippocampus of animals and patients with PTSD [80]. Moreover, astrocyte positive cells were increased in the hippocampus after 1 day following the development of SPS-induced PTSD animal model; these then decreased later [81]. The glutamate and glutamine levels also decrease in the mPFC, suggesting a decreased excitatory activity in the mPFC of SPS-induced PTSD models [82]. D-cycloserine, a partial N-methyl-D-aspartate (NMDA) agonist, enhances fear extinction in animals and patients with PTSD [83,84,85]. Moreover, treatment with NYX-783, the NMDA receptor (NMDAR)-positive modulator, significantly modulated spontaneous recovery and enhanced fear extinction through activation of GluN2B and BDNF in animal models of PTSD [86]. Therefore, the regulation of glutamate levels via NMDAR modulation may be an effective treatment for PTSD.

## 5. Effects of Herbal Medicines on PTSD via Amelioration of Disturbances in the HPA Axis

### 5.1. Bioactive Compounds

Resveratrol is a polyphenolic compound that is widely found in grapevines, peanuts, and pomegranates. It has antioxidant, anti-inflammatory, and anti-diabetic effects, and ameliorates depression, anxiety, and schizophrenia [87,88]. Using the EPM and contextual freezing tests, Zhang et al. demonstrated that resveratrol (20 and 40 mg/kg) decreased anxiety and fear memory in an animal PTSD model. Moreover, resveratrol treatment decreased the CORT, CRH, and ACTH levels in the serum and increased the progesterone and allopregnanolone levels in the PFC and hippocampus of the animal PTSD model; allopregnanolone had a potent inhibitory action on the HPA axis activity [89].

Lee et al. evaluated the efficacy of many traditional medicines and biocompounds using rat models of PTSD. Ginsenoside Rb1 (GRb1) is an active compound in Korean red ginseng (KRG); many studies have shown that KRG and its active compounds exert anxiolytic, anti-depressant, and anti-cognitive impairment effects [90,91,92]. Using the EPM test and the open field test (OFT), Lee et al. showed that GRb1 (30 mg/kg) decreased anxiety-like behaviors. Furthermore, it blocked the SPS-induced increase in the serum CORT levels and increased the expression of hypothalamic neuropeptide Y (NPY) and the hippocampal BDNF mRNA [93].

Ginseng is also rich in ginsenoside Rg1 (Rg1), a steroidal saponin with anti-inflammatory, antioxidant, and anti-apoptotic effects against memory impairment [94]. Wang et al. demonstrated the effects of pretreatment with Rg1 for 7 days in an animal model with footshock- and situation reminder-induced PTSD; Rg1 treatment (5 mg/kg) significantly decreased anxiety symptoms based on the EPM and black and light-box tests. Furthermore, Rg1 treatment significantly decreased the serum CORT and hypothalamus CRH levels [95]. Zhang et al. demonstrated that Rg1 (20 mg/kg) modulated fear extinction impairment (based on the results of the fear conditioning test [FCT]) and depression-like symptoms (based on the results of the OFT, tail suspension test, and forced swimming test [FST]) in an animal model with SPS-induced PTSD. In addition, Rg1 administration ameliorated the levels of hippocampal pro-inflammatory cytokines (TNF-α and IL-1β) and synaptic proteins (PSD95, Arc, GluN2A, and GluA1) [96].

Animal studies have revealed that *Gastrodia elata* Blume has many biological activities, including blood-brain barrier penetration and anti-anxiety and anti-depressant effects. Gastrodin (GAS) is the main compound of *Gastrodia elata* Blume. Lee et al. studied GAS (100 mg/kg) and recovered the duration of immobility in the FST. Furthermore, GAS ameliorated body weight loss and decreased the serum CORT levels, hippocampal NE concentrations, and TH expression. Furthermore, GAS treatment attenuated SPS-induced decreases in the hypothalamic NPY and hippocampal BDNF expressions [97].

Hesperidin (HSD), a flavonoid compound found in citrus fruits, has anti-inflammatory, antioxidant, and sedative properties. Intraperitoneal injection of HSD exerts anti-depressant effects [98]. Using the FST and OFT, Lee et al. demonstrated that HSD (100 mg/kg) decreased depression and anxiety-like behaviors and attenuated fear memory in an SPS-induced PTSD animal model. HSD also increased the sucrose intake and decreased the serum CORT levels in the PTSD model. Furthermore, HSD regulated the serotonergic nervous system and MAO-A activity inhibition and also decreased the levels of TPH1; these demonstrated the potential mechanisms underlying the behavioral effects of PTSD [27].

Soybeans are very popular dietary foods in Asia; one of the major isoflavones in soyabeans is genistein (GEN; 4, 5, 7-trihydroxyisoflavone), which has a wide range of bioactivities. GEN inhibits estrogen- and androgen-mediated signaling pathways in carcinogenesis. Moreover, it exerts anti-inflammatory, antioxidant, and anti-apoptotic effects. Using the Morris water maze (MWM) and object recognition task (ORT) tests, Lee et al. demonstrated that GEN (10 mg/kg) improved memory impairment in an animal PTSD model. Furthermore, GEN treatment regulated the disturbance of HPA axis; GEN decreased the serum CORT levels, increased the 5-HT concentrations in the hippocampus and mPFC, and decreased the 5-HT system through regulation of the 5-HIAA/5-HT and MAO-B levels [99].

Oleuropein (OLE) is the main compound in olive leaves and has several pharmacological properties, including multiple physiological actions and neuroprotective effects. OLE protects against ischemia-induced hippocampal neuronal cell death [100] and ameliorates colchicine-induced cognitive dysfunction [101]. Lee et al. examined whether OLE (100 mg/kg) administration improved cognitive memory in an SPS-induced PTSD animal model; results of the ORT and MWM tests revealed that OLE improved cognitive impairment. Furthermore, it decreased the serum levels of CORT and pro-inflammatory cytokines (TNF-α and IL-1β). OLE-regulated SPS also decreased the expression of BDNF and cAMP response element-binding protein (CREB) in the hippocampus [102].

L-tetrahydropalmatine (THP), extracted from *Corydalis yanhusuo*, has anti-coagulant, anti-nociceptive, antioxidant, anti-viral, and anti-inflammatory effects [103]. Using the EPM test, OFT, and FST, Lee et al. found that THP (50 mg/kg) administration significantly inhibited the reduction in sucrose intake and ameliorated anxiety and depression-like behaviors. Furthermore, THP decreased the serum CORT level; it also increased the NYP level and decreased the CRH level in the PVN of the hypothalamus [104].

Silibinin (SIL), a polyphenolic flavonoid extracted from milk thistle (*Silybum marianum*), has antioxidant, anti-inflammatory, and neuroprotective effects in animal models of neurodegenerative disease [105]. SIL (100 mg/kg) ameliorated depression-like behaviors (based on the results of the FST), anxiety-like behaviors (based on the results of the OFT), and fear memory (based on the results of the FCT). SIL also decreased the serum CORT levels and modulated the serotonin and dopamine system in the amygdala and hippocampus [106].

Curcumin (CUR) is a yellow-pigmented polyphenolic compound extracted from turmeric (*Curcuma longa*), a famous Indian spice. CUR exerts neuroprotective effects in many neuropsychiatric disorders, such as depression, bipolar disorder, and autism [107]. A study noted that CUR (100 mg/kg) ameliorated SPS-induced reduced sucrose preference; furthermore, using the FST, OFT, and FCT, it found that CUR decreased depression, anxiety-like behavior, and fear memory. Furthermore, CUR administration decreased the serum CORT levels, increased the 5-HT levels in the hippocampus and amygdala, and increased TPH-1 mRNA expression in the hippocampus [108].

Umbelliferone (UMB) is widely found in many plants and vegetables, including coriander, carrots, and garden angelica. UMB has antioxidant, anti-inflammatory, analgesic, and sedative properties [109]. In one study, FST and OFT revealed that UMB (60 mg/kg) increased sucrose preference and decreased depression and anxiety-like behaviors. Furthermore, the FCT revealed that UMB treatment decreased fear memory. Moreover, UMB also decreased the serum CORT levels and modulated the serotonin system in the hippocampus and amygdala. In addition, it increased the dopamine levels in the hippocampus [110].

Cannabidiol (CBD), the main component of *Cannabis sativa*, has anti-inflammatory, antioxidant, and neuroprotective effects. Using the OFT, EPM behavior test, and freezing time in a model with footshock-induced PTSD, Pang et al. demonstrated that nasal CBD inclusion of complex temperature-sensitive hydrogels (CBD TSGs, 30 mg/kg) had blocked anxiety-like behaviors. CBD TSGs decreased PTSD-induced neuronal damage and c-fos activation in the PFC, CA1 region, and amygdala, which indicates neuronal excitability. In addition, CBD TSGs decreased the serum TNF-α levels and increased hippocampal 5-HT1A receptor expression in an animal PTSD model. Interestingly, CBD concentration in the blood and brain after intranasal treatment was higher than oral administration [111].

Tetramethylpyrazine (TMP) is an active compound extracted from *Ligusticum wallichii* Franch. It has neuroprotective, antioxidant, anti-apoptotic, and anti-inflammatory effects against neurodegenerative diseases. TMP (40 mg/kg) ameliorated anxiety-like behaviors (based on the results of the EPM test and OFT) and decreased fear memory (based on the results of the FCT). Furthermore, it decreased the serum CORT and ACTH levels. Finally, it increased the 5-HT level in the mPFC and hippocampus, and also increased tryptophan and 5-HIAA mRNA expression in the hippocampus [25].

### 5.2. Herbal Extracts

*Morinda officinalis* (MO) is a well-known traditional medicine that is also used as a nutritional supplement in southern China. MO has anti-stress, anti-depressant, anti-inflammatory, and antioxidant effects. Furthermore, extracts of inulin-type oligosaccharides from MO (IOMO) ameliorate depression [112]. Using the CFT and EPM test, Qiu et al. demonstrated that treatment with IOMO (25 and 50 mg/kg) significantly decreased fear memory and anxiety in an SPS-induced PTSD animal model. IOMO increased allopregnanolone levels in the PFC, hippocampus, and amygdala regions of the brain in a PTSD animal model [113].

KRG is a famous traditional medicine in Korea; fresh roots of 6-year-old ginseng plants are steamed and dried sufficiently. The final product has the same color as that of cherry blossoms [114]. It has many biological activities, such as immune system improvement, alleviation of fatigue, and antioxidant and anti-inflammatory effects. Using the FST, OFT, and FCT, Lee et al. demonstrated that KRG (100 mg/kg) significantly decreased depression-like behaviors and fear memory in an SPS-induced PTSD animal model. Furthermore, KRG decreased the serum CORT levels and increased sucrose preference significantly. Additionally, KRG increased the 5-HT and dopamine levels and decreased the NE levels in the hippocampus and mPFC. KRG also decreased the 5-HIAA/5-HT ratio and the MAO-B concentration in the hippocampus. KRG treatment accordingly modulated the disturbance of HPA axis and the serotonergic system to inhibit depression-like behaviors in a PTSD rat model [115].

*Radix Polygalae* (RP) is a useful traditional medicine in East Asia; it is used in expec-torants, memory enhancers, and sleep aids [116]. The extract blocks scopolamine-induced memory impairment [117] and depression [118]. Shin et al. demonstrated that RP (0.1 mg/kg) attenuated the contextual freezing response but had no effect in an SPS and conditioned fear (SPS-CF)-induced PTSD animal model according to a conditioned fear test. Researchers found that the expression of Bcl-2-associated athanogene (BAG1) was decreased in SPS-CF, which regulates GR activity [119]. Moreover, RP treatment significantly increased BAG1 expression, which is related to the inhibition of fear memory, in an animal PTSD model [120].

The ethanolic extract of *Aralia continentalis Kitagawa* (AC) has sedative, anti-fungal, and anti-inflammatory effects. Using the ORT and the MWM test, Lee et al. found that AC (100 mg/kg) administration decreased the serum CORT levels and increased recognition memory. Furthermore, AC administration increased BDNF and CREB and decreased the levels of pro-inflammatory cytokines (TNF-α and IL-6) in the hippocampus [121].

### 5.3. Herbal Formula

Free and Easy Wanderer Plus (FEWP) is a well-known Chinese traditional formula with anti-depressant and anxiolytic effects. In one study, the EPM and contextual fear tests revealed that treatment with FEWP (10 mg/kg) significantly decreased anxiety and fear memory. It also increased the allopregnanolone levels in the prefrontal cortex, hippocampus, and amygdala following exposure to SPS [122].

Anshen Dingzhi prescription (ADP) is an important formula for the treatment of neurodegenerative and mental disorders. In one study, the EPM test, OFT, and fear memory test revealed that ADP (18.4 and 36.8 mg/kg) decreased anxiety and fear memory in an SPS-induced PTSD animal model. Hematoxylin and eosin staining also revealed that ADP has neuroprotective effects in the hippocampal CA1 region. Furthermore, researchers found that ADP reduced PTSD-like behaviors by regulating synaptic protein levels [123].

## 6. Conclusions

**Figure 1:**
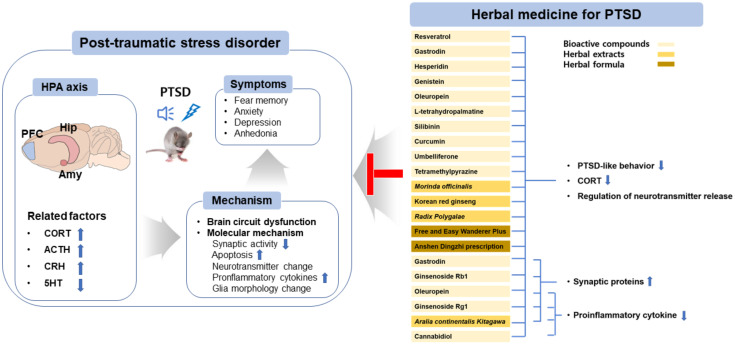
Overview of the pharmaceutical intervention based on herbal medicine for treating PTSD. Abbreviations: hypothalamic–pituitary–adrenal axis (HPA axis); prefrontal cortex (PFC); hippocampal (Hip); amygdala (Amy); corticotropin releasing hormone (CRH); adrenocorticotropic hormone (ACTH); corticosterone (CORT); serotonin (5-hydroxytryptamine (5-HT)); brain-derived neurotrophic factor (BDNF); cAMP response element-binding protein (CREB).

In this review, we have provided an overview of the molecular mechanisms of PTSD and have summarized the effects of herbal medicines based on the HPA axis in animal PTSD models (Table 1, Figure 1). PTSD is known to be comorbid with depression, anxiety, and alcohol abuse; therefore, the mechanisms are very complex and diverse. Among these, disturbances in the HPA axis play the main role in PTSD development, maintaining homeostasis in the central and peripheral nervous systems. Importantly, HPA axis modulators, such as decreasing CORT or GR antagonists, may be used to target PTSD. In addition, herbal medicines with multitarget effects could be applied to diseases with multiple mechanisms. We found that many active compounds, extracts, and formulas have good effects on PTSD-related behaviors, such as fear memory, anxiety, and depression. Therefore, herbal medicines could be useful candidates for treating psychiatric disorders; they can also serve as good candidates for the development of novel and promising natural drugs. Sertraline and paroxetine are very expensive, have serious side effects, and require more treatment time in patients with PTSD. We suggest that a combination of Western and herbal medicines can reduce the required concentrations of sertraline and paroxetine, which may be a good choice for PTSD treatment.

Our review has some limitations. We searched for research articles on animal models with SPS-induced and footshock-induced PTSD; articles dealing with social defeat stress-induced PTSD and predator stress-induced PTSD were excluded. Furthermore, the mechanisms and efficacy of herbal medicines differ between animals and humans; therefore, human clinical trials should be conducted in the future. It is also important that herbal medicinal formulations be optimized for obtaining the best therapeutic effects. Another limitation is that most articles on the efficacy of herbal medicines against PTSD were reported by one group.

In conclusion, herbal medicines have the potential to protect against or treat psychological diseases, such as PTSD.

## Figures and Tables

**Table 1:** Summary of the effects of herbal medicine used in PTSD animal model based on HPA axis.

	Herbal Medicine	Model	Treatment (Concentration/ Period)	Action Mechanism	Author (Year) Reference
	Resveratrol	FS	20, 40 mg/kg (i.g.) for 17 days	∙ Anti-anxiety and anti-fear memory behavior ∙ Decreased HPA- axis hormones (CORT, CFH, ACTH) ∙ Increased progesterone and allopregnanolone	Zhang (2017) [89]
	Ginsenoside Rb1 (GRb1)	SPS	30 mg/kg (i.p.) for 14 days	∙ Anti-anxiety behavior ∙ Increased NPY, decreased TH, increased BDNF mRNA ∙ Decreased CORT	Lee (2015) [93]
	Ginsenoside Rg1 (Rg1)	FS	5 mg/kg (p.o.) for 7 days	∙ Anti-anxiety behavior ∙ Decreased CORT and CRH	Wang (2015) [95]
	Ginsenoside Rg1 (Rg1)	SPS	20 mg/kg (i.p.) for 14 days	∙ Anti-depression behavior, increased fear extinction ∙ Decreased hip pro-inflammatory cytokines (IL-1β and TNF-α) ∙ Increased synaptic proteins (PSD95, Arc, GluN2A, GluA1) in hipo	Zhang (2021) [96]
	Gastrodin (GAS)	SPS	100 mg/kg (i.p.) for 14 days	∙ Anti-depression behavior ∙ Increased body weight ∙ Decreased CORT, NE, and TH ∙ Increase NPY and BDNF	Lee (2016) [97]
Bioactive compounds	Hesperidin (HSD)	SPS	100 mg/kg (i.p.) for 14 days	∙Anti-depression, anti-anxiety, and anti-fear memory ∙ Increased sucrose intake ∙ Decreased CORT ∙ Increased 5-HT in the hip and amy ∙ Decreased 5-HIAA/5-HT and MAO-A activity ∙ Increased DA in the hip ∙ Increased TPH1 in the hip	Lee (2021) [27]
	Genistein (GEN)	SPS	10 mg/kg (i.p.) for 14 days	∙ Increased cognitive memory ∙ Decreased CORT ∙ Increased 5-HT level in the hip and mPFC ∙ Decreased 5-HIAA/5-HT ratio and MAO-B activity	Lee. (2020) [99]
	Oleuropein (OLE)	SPS	10 mg/kg (i.p.) for 14 days	∙ Improved cognitive memory ∙ Decreased CORT ∙ Increased BDNF and CREB in the hip ∙ Decreased TNF-α and Il-1β	Lee. (2018) [102]
	L-tetrahydropalmatine (THP)	SPS	50 mg/kg (i.p.) for 14 days	∙ Anti-anxiety and anti-depression-like behaviors ∙ Decreased CORT ∙ Increased NYP and decreased CRH in the hypo	Lee. (2020) [104]
	Silibinin (SIL)	SPS	100 mg/kg (i.p.) for 14 days	∙ Anti-anxiety, anti-depression-like behavior, and anti-fear memory ∙ Decreased CORT ∙ Increased 5-HT in the hip and amy ∙ Decreased 5-HIAA ∙ Increased DA in the hip ∙ Increased TPH-1 mRNA in the hip	Lee. (2020) [106]
	Curcumin (CUR),	SPS	100 mg/kg (i.p.) for 14 days	∙Anti-anxiety, anti-depression-like behaviors, and anti-fear memory ∙ Decreased CORT ∙ Increased 5-HT in the hip and amy ∙ Increased TPH-1 mRNA in the hip	Lee, (2018) [108]
	Umbelliferone (UMB)	SPS	60 mg/kg (i.p.) for 14 days	∙ Anti-anxiety, anti-depression-like behaviors, and anti-fear memory ∙ Decreased CORT ∙ Increased 5-HT in the hip and amy ∙ Decreased 5-HIAA/5-HT ratio and MAO-A ∙ Increased DA in the hip	Lee, (2019) [110]
Bioactive compounds	Cannabidiol (CBD)	FS	30mg/kg (nasal administration) for 10 days	∙ Anti-anxiety and anti-fear memory ∙ Neuroprotection effects ∙ Decreased c-fos activation in the PFC, hip CA1 and amy ∙ Decrease TNF-α ∙ Decrease 5-HT1A receptor in the hip	Pang (2021) [111]
	Tetramethylpyrazine (TMP)	SPS	40 mg/kg (i.p.) for 14 days	∙ Anti-anxiety and anti-fear memory ∙ Decreased CORT and ACTH ∙ Increased 5-HT in the mPFC and hip ∙ Increased TRP and 5-HIAA mRNA in the hip	Lee, (2018) [25]
	*Morinda officinalis* (MO)	SPS	25, 50 mg/kg (i.p.) for 13 days	∙ Anti-anxiety and anti-fear memory ∙ Increased allopregnanolone biosynthesis in the hip, PFC, and amy	Qiu (2016) [113]
Herbal extracts	Korean red ginseng (KRG)	SPS	100 mg/kg (i.p.) for 14 days	∙ Anti-depression and anti-fear memory ∙ Decreased CORT ∙ Increased sucrose performance ∙Increased 5-HT in the hip and mPFC, and decreased 5-HIAA/5-HT ∙ Decreased NE and increased DA in the hip ∙ Decreased TPH-1, TPH-2, and MAO-B the hip	Lee (2020) [115]
	*Radix polygalae* (RP)	CF + SPS	0.1 mg/kg (p.o.) for 13 days	∙ Anti-fear memory ∙ Increased BAG1 in the hip	Shin (2018) [120]
	*Aralia continentalis Kitagawa* (AC)	SPS	100 mg/kg (i.p.) for 21 days	∙ Increase recognition memory ∙ Decreased CORT ∙ Increased BDNF and CREB in the hip ∙ Decreased TNF-α and IL-6 in the hip	Lee, (2019) [121]
Herbal formula	Free and Easy Wanderer Plus (FEWP)	SPS	5, 10 mg/kg (drinking water) for 14 days	∙ Anti-anxiety and anti-fear memory ∙ Increased allopregnanolone in the PFC, hip, and amy	Qiu (2015) [122]
	Anshen Dingzhi prescription (ADP)	SPS	18.4, 36.8 mg/kg (i.g.) for 14 days	∙ Anti-anxiety and anti-fear memory ∙ Neuroprotective effect ∙Regulated synaptic proteins (PSD95, BDNF-TrkB signaling pathway, μ-calpain/PTEN-AKT-mTOR signaling pathway)	Yang (2022) [123]

Abbreviations: single prolonged stress (SPS); conditioned fear (CF); footshock (FS); intragastric administration (i.g.); per os (p.o.); medial prefrontal cortex (mPFC); hippocampus (hip); amygdala (amy); hypothalamus (hypo); hypothalamic–pituitary–adrenal axis (HPA axis); paraventricular nucleus (PVN); corticotropin releasing hormone (CRH); adrenocorticotropic hormone (ACTH); corticosterone (CORT); neuropeptide Y (NPY); tryptophan (TRP); tyrosine hydroxylase (TH); tryptophan hydroxylase (TPH); serotonin (5-hydroxytryptamine (5-HT)); 5-Hydroxyindoleacetic acid (5-HIAA); monoamine oxidase (MAO), norepinephrine (NE); dopamine (DA); interleukin 1 beta (IL-1β); tumor necrosis factor-α (TNF-α); interLeukin-6 (IL-6); brain-derived neurotrophic factor (BDNF); cAMP response element-binding protein (CREB); postsynaptic density protein 95 (PSD95); activity-regulated cytoskeleton-associated protein (Arc); NMDA receptor subunit 2A (GluN2A); AMPA receptor subunit 1 (GluA1); tropomyosin receptor kinase B (TrkB); protein kinase B (AKT); mammalian target of rapamycin (mTOR); Phosphatase and tensin homolog (PTEN).

## Data Availability

Data are contained in the article.
